# The Genetic Effect on Muscular Changes in an Older Population: A Follow-Up Study after One-Year Cessation of Structured Training

**DOI:** 10.3390/genes11090968

**Published:** 2020-08-21

**Authors:** Lingxiao He, Evelien Van Roie, An Bogaerts, Sabine Verschueren, Christophe Delecluse, Christopher I. Morse, Martine Thomis

**Affiliations:** 1 Department of Movement Sciences, Physical Activity, Sports & Health Research Group, KU Leuven, 3001 Leuven, Belgium; lingxiao.he@hotmail.com (L.H.); evelien.vanroie@kuleuven.be (E.V.R.); an.bogaerts@kuleuven.be (A.B.); christophe.delecluse@kuleuven.be (C.D.); 2 Department of Sport and Exercise Sciences, Manchester Metropolitan University, Oxford Road, Manchester M15 6BH, UK; c.morse@mmu.ac.uk; 3 Department of Musculoskeletal Rehabilitation, Rehabilitation Sciences Research Group, KU Leuven, 3001 Leuven, Belgium; sabine.verschueren@kuleuven.be

**Keywords:** genetic predisposition score, muscle, older adults, cessation of structured training

## Abstract

Older adults lose muscle mass and strength at different speeds after the cessation of physical exercise, which might be genotype related. This study aimed to explore the genetic association with changes in muscle mass and strength one year after the cessation of structured training in an older population. Participants (*n* = 113, aged between 61 and 81 years) who performed one-year of combined fitness (*n* = 44) or whole-body vibration (*n* = 69) training were assessed one year after the cessation of the training. Whole-body skeletal muscle mass and knee strength were measured. Data-driven genetic predisposition scores (GPSs) were calculated and analysed in a general linear model with sex, age, body mass index and post-training values of skeletal muscle mass or muscle strength as covariates. Forty-six single nucleotide polymorphisms (SNPs) from an initial 170 muscle-related SNPs were identified as being significantly linked to muscular changes after cessation. Data-driven GPSs and over time muscular changes were significantly related (*p* < 0.01). Participants with higher GPSs had less muscular declines during the cessation period while data-driven GPSs accounted for 26–37% of the phenotypic variances. Our findings indicate that the loss of training benefits in older adults is partially genotype related.

## 1. Introduction

The process of ageing is commonly accompanied by progressive loss in skeletal muscle mass and muscle strength [1]. A 3-year follow-up study of Goodpaster et al. [2] on older adults aged 70–79 years has revealed a 1% annual loss in leg lean mass with muscle strength decreasing three times faster than muscle mass. These declines in knee extensor strength and thigh muscle mass are associated with increased risk of mobility loss in the older population [3]. Consequently, physical performance and quality of life in older adults are largely affected by functional and structural alterations in ageing muscles [4].

Exercise, such as fitness training and whole-body vibration (WBV) training, has been well established as an effective non-pharmacological method to counteract muscle degeneration in older adults [5,6,7,8]. Besides the large number of studies on exercise benefits for ageing muscle, many studies have also focused on the lasting benefits of exercise by describing the loss of muscle strength and size following exercise cessation, termed as “detraining”. For example, the lasting of training benefits is training intensity dependent. In the study of Fatouros et al. [9], strength and mobility gains of older men who received a high intensity resistance training lasted longer than those who trained at a low intensity. Moreover, muscle size and muscle strength do not decrease at the same speed during detraining. Older women who completed 12 weeks of resistance training retained a 12% gain in knee extensor strength after 3-month detraining, while the muscle volume of knee extensors had already dropped back to baseline levels [10]. These muscular decreases during detraining are multifactorial. Composition changes such as fat infiltration [11] and reduced cross-sectional area of type I and type II fibres [12], morphological alterations like decreased pennation angle and fascicle length [13], neural control [14] and hormone [15] changes have all been reported to be associated with decreased muscle strength in the detraining period. To some extent, the detraining period in older adults can also be regarded as an ageing process and therefore, some ageing muscle-related mechanisms, such as myofibre denervation [16], atrophy of type II myofibres [17], decreased mitochondrial enzyme activity [18] and reduced synthesis rates of myosin heavy chain [19], might also be linked to the lasting of exercise benefits. However, the association between muscle-related genetic makeup and muscular changes after the cessation of structured training is poorly understood.

Multiple genetic variants have been associated with inter-individual difference within the training response among young adults [20] and older women [21]. As reported in the study of Delmonico et al. [21], older women with *ACTN3* R577 XX genotype had higher baseline knee extensor power than R-homozygous carriers, while the latter had greater improvement after 10 weeks of strength training. To analyse a polygenic connection with muscular phenotypes, an approach of genetic predisposing score (GPS), which is calculated based on muscle-related genotypes, has been used in multiple genetic studies. There are several methods to calculate a GPS. Briefly, a GPS can be calculated either by directly adding equally-weighted muscle-favourable alleles (e.g., total GPS [22] and data-driven GPS [23,24]) or by summing the weights of selected favourable alleles, the weight of which is determined by statistical models (e.g., weights based on explained variances of a phenotype [25], effect sizes [26] and elastic net coefficients [27]). Despite the different calculation methods, Charlier’s study [27] showed that those GPSs explained a similar variance (around 5%) of muscle mass and shared nearly 19% of favourable gene variants based on the same candidate gene pool. The GPS has been adopted to explain variances in thigh muscle mass and knee strength after cardiac rehabilitation training [23], to explore muscular changes with ageing [27], and to evaluate athletic status [28]. Given our previous findings that GPS explained 14% and 27% of exercise-induced increases in muscle mass and muscle strength [24], respectively, we hypothesise a genetic association with muscular changes after the cessation of a structured training intervention. Therefore, the purpose of this research is to study the genetic effect on muscular changes after one-year of exercise cessation in an older population.

## 2. Materials and Methods

### 2.1. Participants

**Figure 1:**
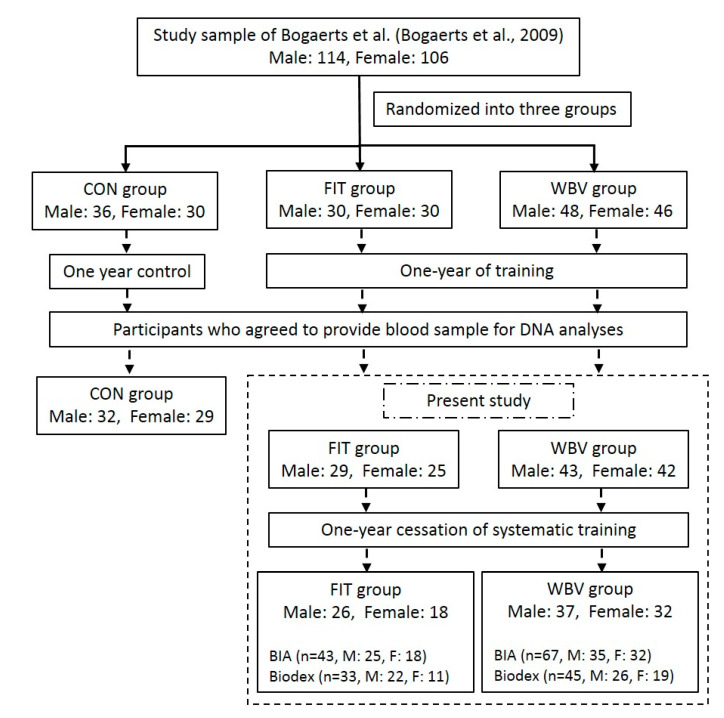
Flowchart of participants in present study. CON: control; FIT: combined fitness; WBV: whole-body vibration; BIA: bioelectrical impedance analysis.

Participants, aged between 61 and 81 years, were originally recruited in an exercise intervention study of Bogaerts et al. in 2009 [29]. Older adults with physical disorders that might affect exercise performance or with any training experience in the past two years were excluded. In the study, participants were randomly assigned into a control (CON) group, a combined fitness (FIT) group or a WBV group (Figure 1). The training intervention lasted for one year and the participants were not aware of a follow-up test. One year after the training program, participants who had provided blood samples for genotyping in the exercise (FIT or WBV) groups, were contacted for a follow-up test. Since our previous study has reported the adaptive changes of muscle mass and muscle strength induced by exercise [24], this study mainly focused on the muscular changes in these exercise groups after the cessation. Noticeably, the study of Bogaerts et al. [29] only included the participants who fully completed the training program. In our study, we included participants who had more than 60% of attendance during the training and completed at least one of the follow-up measurements (Figure 1). This ensured a comparatively large sample size for further genetic study. This study was approved by the Ethics Committee Research UZ/KU Leuven (Project identification code: ML2508, Date of approval: 22 December 2003) and all the participants were asked to sign an informed consent form.

### 2.2. Training Protocols

The training programs (Table S1) have been described in detail in the study of Bogaerts et al. [29]. Briefly, participants in the exercise groups trained three times per week on non-consecutive days for one year. The training program for the FIT group was designed following the ACSM guidelines for older adults exercise prescription [30], which consisted of aerobic, resistance, balance and flexibility exercises. Participants in the WBV group were instructed to perform static and dynamic leg exercises on vibration platforms (Power Plate, Amsterdam, The Netherlands). The training programs were performed at Leuven University’s training centre under the guidance and supervision of qualified health and fitness instructors. Participants in the CON group were advised to maintain their lifestyle and to not engage in any new physical activity.

### 2.3. Genotyping

A 4.5 mL venous blood sample was collected from each participant using an EDTA-coated tube. DNA was extracted using the chemagic Magnetic Separation Module I (chemagic MSM I, PerkinElmer Inc., Waltham, MA, USA). Genotyping was completed with GoldenGate assay (Illumina, Inc., San Diego, CA, USA) following the protocols of the manufacturer [31]. Single nucleotide polymorphisms (SNPs) that were reported to be associated with the development or regulation of muscle function or muscle growth were selected based on published articles (up to August 2014) and expression quantitative trait loci (eQTL) analysis. In total, 224 muscle-related SNPs (Table S2) were genotyped from each blood sample. From the genotyping results, 12 SNPs had a detection success rate of less than 80%; 3 SNPs showed the same genotypes among all the participants. Linkage disequilibrium (LD) analysis was performed and 58 SNPs had high LD (absolute correlation coefficient greater than 0.8) as 19 subgroups. Within these subgroups, the SNP with the largest number of correlated SNPs or published references was selected as a representative for each subgroup (Table S3). In the end, 54 SNPs were excluded from the initial SNP pool and 170 SNPs were kept for further analyses.

### 2.4. Parameter Measurements

Electrical resistance of the body was measured by bioelectrical impedance analysis (BIA) using Bodystat 1500MDD (Bodystat Ltd., Douglas, UK). Skeletal muscle mass (SMM) was estimated using the following equation which was developed by Janssen et al. [32]: SMM (kg) = (Ht^2^/R × 0.401) + (sex × 3.825) + [age × (−0.071)] + 5.102 where Ht stands for height in centimetres; R stands for BIA resistance in ohms; in sex, men = 1 and women = 0; age is in years. SMM calculated by this equation showed validity among older adults with a standard error of estimate of 2.7 kg (9%) [32].

Isometric, isotonic and isokinetic knee extensor strength was tested using Biodex Medical System 3 dynamometer (Biodex Company, New York, NY, USA). Participants were asked to complete a 5-min warm up on a free-loaded cycle ergometer followed by two practice trials on the dynamometer to ensure some familiarization. In the actual tests, each of the following protocols were performed twice and the maximum value of each protocol was recorded for further analyses.

Isometric test: peak torque of isometric knee extension was measured at a knee flexion angle of 60° (PT_IM60_ in Nm, 0° representing full extension) with a duration of 5 s. Maximal isometric strength at the flexion angle of 90° was also recorded for load setting in the isotonic test.

Isotonic test: the isotonic test included 3 sets of ballistic knee extension movements with a load of 20% of the peak isometric strength obtained at the knee flexion angle of 90°. Starting at the knee flexion angle of 90°, participants were asked to extend their legs as fast as possible until they achieved the knee flexion angle of 20°. Peak velocity (PV_IT20_ in °/s) was recorded for further analyses.

Isokinetic test: participants performed isokinetic knee extension and flexion movements at two different speeds. The first measurement required participants to complete four repetitions at a low velocity of 60°/s. The second measurement consisted of six repetitions at a higher velocity of 240°/s. Peak torque of knee extensors at 60°/s (PT_IK60_ in Nm) and at 240°/s (PT_IK240_ in Nm) were recorded and further analysed.

### 2.5. Statistical Analyses

All data are reported as mean ± standard deviation (SD) and were analysed using SAS statistical software version 9.4 for Windows (SAS Institute Inc., Cary, NC, USA). Since muscle mass and muscle strength can be affected by multiple factors, the effect of a single gene on muscle is rather limited. Therefore, an accumulative effect of multiple gene variants was hypothesized in this study. To stay consistent with the data-driven GPS calculation method used in our previous studies [23,24,27], alleles associated with less muscular decreases over time were regarded as predisposing alleles and were equally weighted as 1. Stepwise regression analysis, with an entry/exit significance of 0.1/0.05, was used in the selection of SNPs (from a SNP pool of 170) that were significantly related to relative change of each muscular phenotype after the cessation. Genetic predisposition score (GPS) of each participant was calculated by adding up the weight of each phenotype-driven genotype. For example, using stepwise regression, allele G of SNP rs3762546 in gene *MSTN* was found to be favourable for ∆PT_IM60_ (i.e., being associated with less PT_IM60_ decline) after one-year of cessation. Thus, the genotype score of rs3762546 was calculated based on the number of G allele: GG = 2, CG = 1 and CC = 0. ∆PT_IM60_-driven GPS in a participant was calculated by summing up scores of all the SNPs that were found significantly related to corresponding phenotypes.

Comparisons between the FIT and the WBV groups at post-training and one-year follow-up tests were made by two-way analysis of variance (ANOVA) with sex and group as factors. Bonferroni method was applied as post-hoc test. The same ANOVA was also completed in the comparisons of relative changes of muscular phenotypes after one-year of exercise cessation. To compare the value of each muscular phenotype between post-training and follow-up tests, repeated measures ANOVA was made with sex and group as factors. A *p* value of 0.05 was set as the level of significance. The predictive value of GPS on relative changes of muscular parameters was evaluated by general linear model (GLM) with age, sex, body mass index (BMI) and corresponding post-training muscle values as covariates.

## 3. Results

### 3.1. Descriptive Data and Relative Changes at Post-Training and Follow-Up Tests

Descriptive data of muscular phenotypes in the FIT and the WBV groups are presented in Table 1. Between-group comparisons showed that participants in the FIT and the WBV groups were not different for muscle mass and muscle strength at both post-training and follow-up tests (*p* > 0.05). By comparisons between post-training and one-year follow-up test, significant increases in BMI were found for both exercise groups (*p* < 0.01) one year after the cessation of structured training. Moreover, PV_IT20_ (*p* < 0.01), PT_IK60_ (*p* = 0.02) and PT_IK240_ (*p* < 0.01) decreased significantly in both exercise groups. Time × sex, time × group or time × sex × group interactions were non-significant for all phenotypes.

### 3.2. Associations of GPS with Relative Muscular Changes after One-Year Cessation of Structured Training

Since no significant differences were found in relative changes between the FIT and the WBV groups, values of the two groups were analysed together for the selection of data-driven SNPs and the evaluation of genetic influence on muscular changes after the one-year cessation of structured training. Muscular phenotype-driven SNPs are presented in detail in Table S4, in which we showed that unlike many genes that contributed only one SNP to muscular changes, more than one SNP was identified in gene *ACVR1B*, *ATP1A2*, *MTHFR* and *MTRR*, respectively. Furthermore, rs2251375 in *H19*, rs3741211 in *IGF2*, rs2390760 in *METTL21C*, rs3762546 in *MSTN*, rs1805087 in *MTR*, rs327575 and rs97713 in *MTRR*, and rs4790881 in *SMG6* were found to be linked with more than one change in muscular parameters (Table S4). Yet, no SNP was found to be associated will all the muscular parameters.

GPS was calculated by summing up the weight of predisposing SNPs. The results of GLM are presented in Table 2. These results showed that data-driven GPS was closely associated with changes in muscular phenotypes one year after the cessation of a structured training intervention (*p* < 0.01). Noticeably, GPS accounted for similar variances (from 26% to 37%) in muscle mass and muscle strength changes during the cessation period. Increasing the data-driven GPS with one predisposing allele is associated with 2.09% to 4.53% less decreases in SMM, PT_IM60_, PV_IT20_, PT_IK60_ and PT_IK240_ after one year.

**Figure 2:**
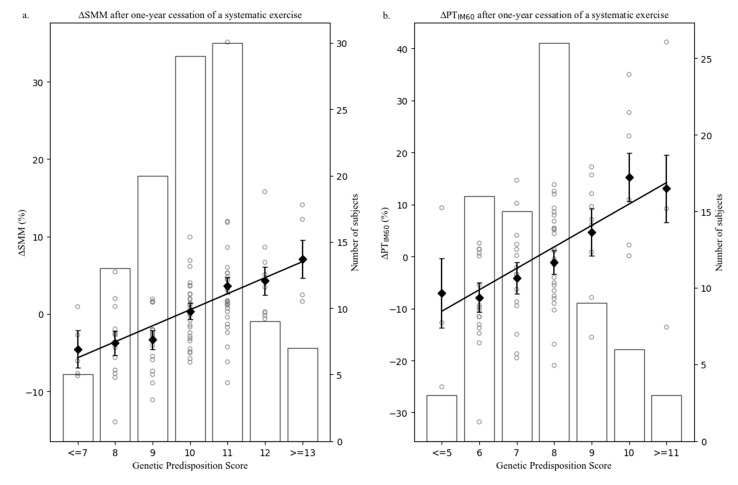

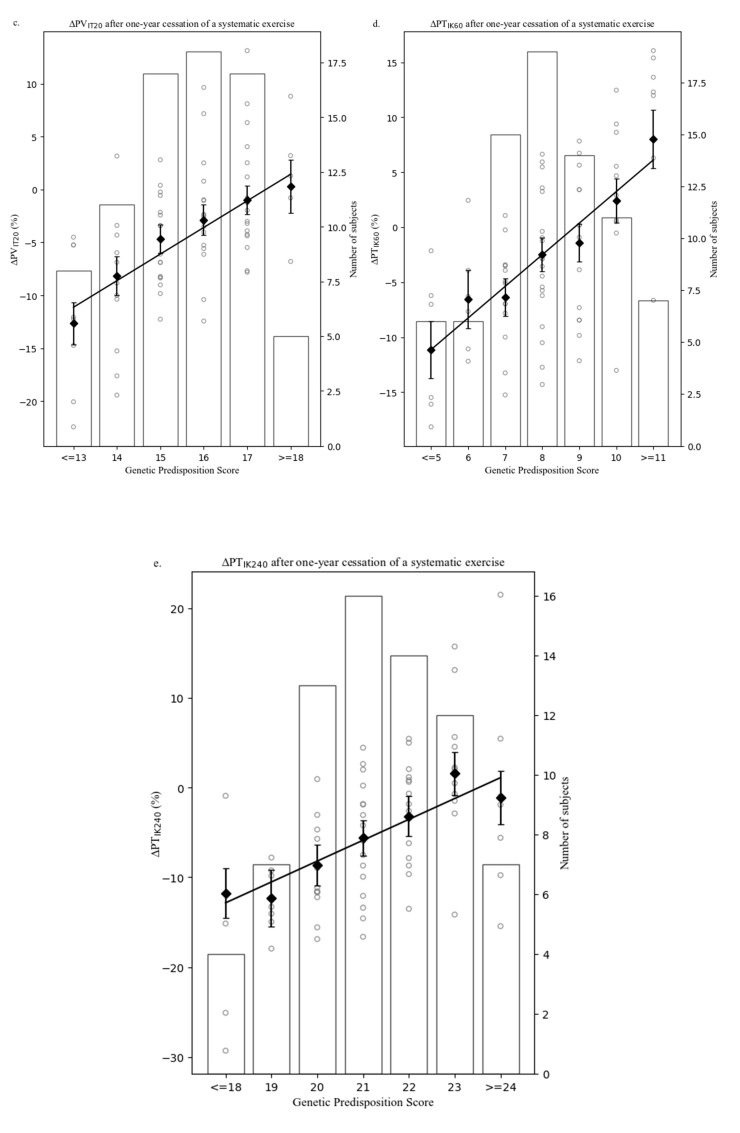
Distribution of GPS and its linear regression model with muscular phenotype changes after one-year cessation of a structured training intervention. (**a**) Linear regression between genetic predisposition score (GPS) and relative change of skeletal muscle mass (∆SMM) in the exercise groups (fitness (FIT) and whole-body vibration (WBV)) after one-year cessation of a structured training regime (adjusted for age, sex, BMI and corresponding post-training value). GPS is calculated based on 9 single nucleotide polymorphisms (SNPs) from 9 genes (rs4870044 in *ESR1*, rs11549465 in *HIF1A*, rs3741211 in *IGF2*, rs7924316 in *IGF2AS*, rs2390760 in *METTL21C*, rs3762546 in *MSTN* and rs97713 in *MTRR*, rs2229139 in *RYR1*, and rs4790881 in *SMG6*). Individual ∆SMM values (%) are presented on the left *y*-axis. The trend line shows the relation between GPS and ∆SMM. Least square means of ∆SMM in each GPS is presented as dot with standard errors presented as error bar. Distribution of participants in each GPS is presented in the histogram with number of participants on the right *y*-axis. Scatterplot is used to present the distribution of ∆SMM in each GPS group. (**b**) Linear regression between GPS and relative change of peak isometric knee extension torque at a knee flexion angle of 60° (∆PTIM60) after one-year cessation of a structured training programme (adjusted for age, sex, BMI and corresponding post-training value). GPS is calculated based on 7 SNPs from 7 genes (rs2296383 in *CACNA1S*, rs8111989 in *CKM*, rs689 in *INS*, rs2390760 in *METTL21C*, rs3762546 in *MSTN*, rs327575 in *MTRR*, and rs28357094 in *SPP1*). (**c**) Linear regression between GPS and relative change of peak velocity of isotonic knee extension (PV_IT20_) after one-year cessation of a structured training intervention (adjusted for age, sex, BMI and corresponding post-training value). GPS is calculated based on 13 SNPs from 11 genes (rs3733890 in *BHMT*, rs6107853 in *BMP2*, rs1800169 in *CNTF*, rs4511463 in *GSC*, rs2251375 in *H19*, rs3741211 in *IGF2*, rs11121828 in *MTHFR*, rs1805087 in *MTR*, rs97713, rs1801394 and rs162031 in *MTRR*, rs1800470 in *TGFB1*, and rs1483246 in *ZNF804A*). (**d**) Linear regression between GPS and relative change of peak torque of isokinetic knee extension at 60°/s (PT_IK60_) after one-year cessation of structured training (adjusted for age, sex, BMI and corresponding post-training value). GPS is calculated based on 9 SNPs from 8 genes (rs2854248 in *ATP1A2*, rs10883631 in *FN1*, rs17727841 in *IGF1*, rs2390760 in *METTL21C*, rs1801133 in *MTHFR*, rs327575 and rs7703033 in *MTRR*, rs4790881 in *SMG6*, and rs10497520 in *TTN*). (**e**) Linear regression between GPS and relative change of peak torque of isokinetic knee extension at 240°/s (PT_IK240_) after one-year cessation of structured training (adjusted for age, sex, BMI and corresponding post-training value). GPS is calculated based on 18 SNPs from 14 genes (rs746434 and rs10783485 in *ACVR1B*, rs12721026 in *APOA1*, rs1016732 in *ATP1A2*, rs3797297 in *FST*, rs2251375 in *H19*, rs2919358 in *KBTBD13*, rs1137101 in *LEPR*, rs3762546 in *MSTN*, rs1476413 and rs1009592 in *MTHFR*, rs1805087 in *MTR*, rs10475399, rs326123 and rs9313211 in *MTRR*, rs4950877 in *MYOG*, rs4253778 in *PPARa*, and rs142196418 in *RIMS1*).

GPS distribution of participants and linear models between GPS and over time changes of muscular phenotypes are presented in Figure 2. GPS was categorized with no less than three participants in each group. As shown in Figure 2a–e, participants with higher GPS had less decreases in muscle mass and muscle strength after one-year cessation of a structured training regime.

## 4. Discussion

### 4.1. Are Gene Variants Related to Muscular Changes after the Cessation of a Structured Training Intervention?

Using the methods of stepwise regression and data-driven GPS, this study analysed the overall genetic effect on muscular changes after one-year cessation of a structured training programme in an older group. From a 170-SNP pool, 46 SNPs of 32 genes (Table S4) were found to be closely associated with muscular changes. GLM results showed that participants with higher GPSs (more favourable alleles) had less decreases in muscle mass and strength after the cessation of training. These models suggested that genetic makeup was associated with inter-individual variance in muscular phenotypes despite that some of the phenotypes (i.e., SMM and PT_IM60_) did not show significant decline after one year, and data-driven GPSs explained 26% to 37% of the variances of these muscular changes during the cessation. Therefore, although a phenotype can show non-significant time-related changes, with large inter-individual variability underlying this finding, the GPS was able to account for a substantial part of these inter-individual changes over time.

The set-up of multi-gene variants and an exercise cessation background makes it difficult to compare our results with other studies. To our knowledge, there is presently no research among older adults regarding the genetic influence on muscular changes following a cessation of training, with limited research investigating the genetic influence on muscular adaptations resulting from exercise intervention. A cross-sectional study carried out by Charlier et al. [27] among 565 Flemish Caucasians (aged 19–73 years) showed that 4.6% to 6.6% of variances in muscle mass and muscle strength could be explained by data-driven GPS. Such limited degrees of explainable variance by GPS might be due to the wide age range in which many non-genetic factors can affect muscular phenotypes in the long term. Therefore, when restricting the set-up to a shorter age range, an increased role for GPS (as what we have found in this study) can be observed. The degree of genetic variation contributing to muscular changes after the cessation of exercise (26% to 37%) are similar to those reported for responses to exercise interventions. With a set of 54 SNPs, data-driven GPS-explained 6% to 26% of variances in knee extension strength and muscle size adaptations after a 3 months of training among coronary artery patients [23]. Our previous study in the same study population also found that data-driven GPS accounted for 14% and 27% of the variances in ∆SMM and ∆PT_IM60_, respectively, after a one-year exercise intervention [24].

In addition, the present study found a few SNPs that were previously reported to be associated with exercise-induced muscular gains. Some of those SNPs even contributed to the change in the same phenotype. Based on our results, the A allele in rs1016732 from gene *ATP1A2* was associated with more decrease in PT_IK240_, which is a parameter of endurance strength, after training cessation. This finding is in line with the study of Sarzynski et al. [33], who reported that people with rs1016732 minor allele (i.e., allele A) would demonstrate more decrease in exercise test duration over a 20-year period. Furthermore, our team have previously reported that *METTL21C* rs2390760 (with C as the favourable allele) and *MSTN* rs3762546 (with G as the favourable allele) were significantly related to increased muscle mass (∆SMM) after WBV and FIT training [24] while these SNPs were also closely associated with the one-year cessation-related SMM change in this study. However, in the present study, allele G was found as a favourable allele in SNP rs2390760 and allele G remained as the favourable allele in SNP rs3762546. This suggests that carriers of the C allele in *METTL21C* rs2390760 are more susceptible to exercise than G allele carriers while allele G in *MSTN* rs3762546 is predisposed for the adaption of muscle mass in exercise as well as its maintenance after the cessation.

### 4.2. What Kind of Genes Are Related to Muscular Alterations after the Cessation of Exercise?

Although the validation on datasets with other older adults still remains to be tested, our findings suggest some representative variants out of a large SNP set that are significantly related to muscular changes after exercise cessation. Based on the categories in Table S2, among the genes that had significant associations with muscular changes in this study, three genes are involved in DNA methylation, three genes are related to hormone expression or its receptor, nine genes encode for growth/differentiation factors, nine genes are metabolism-related, seven genes contribute to muscle/bone structure and three genes are involved in neural control.

The discovery of a contribution of gene *MTHFR*, *MTR* and *MTRR* to muscular changes indicates the involvement of DNA methylation after the cessation of exercise. DNA methylation is one of the mechanisms in epigenetic processes, which regulates gene expression without entailing a change in the DNA sequence [34]. Generally, hypermethylation in promoter regions will repress transcriptions of corresponding genes while hypomethylation will reactivate them. Recent studies have shown that methylation changes can be induced by exercise. In the study of Barrès et al. [35], muscle biopsies were collected 20-min after an acute aerobic capacity test and hypomethylation was found in promoter regions of several metabolism-related genes (*PGC-1α*, *PDK4* and *PPAR-δ*). Meanwhile, hypomethylation also took place in some genes (*BICC1*, *STAG1*, *GRIK2* and *TRAF1*) after both a single bout and a 7-week resistance training program, and returned to baseline levels after cessation of the 7-week training [36]. In our present study, we found that genetic variation in *MTHFR*, *MTR* and *MTRR* genes, which encode for corresponding enzymes that regulate the methylation circle [37], might play a role in altered methylation during the cessation period. Therefore, it is likely that a DNA hypomethylation-favourable gene might be associated with a better response towards training as well as a longer maintenance of the gains when structured training stops.

Genes related to hormone expression, muscle growth/differentiation, metabolism or muscle/bone structure have been linked to physical performances by many studies [20,38,39]. *PPARa* intron 7 (rs4253778) G/C polymorphism has been reported as exercise-oriented with a high frequency (80%) of GG genotype existing among endurance athletes. Further biopsy analysis showed a higher percentage of slow-twitch fibres in GG carriers when compared with the CC counterpart [40]. Similarly, in the aspect of one-year cessation-related muscular changes among the participants in our study, allele G was found favourable (with less decrease) for the change of dynamic muscle strength at a high contraction speed (∆PV_IK240_).

In the domain of neural factors, this study identified three SNPs from three genes, among which is the gene *CNTF*. Encoding for ciliary neurotrophic factors, the rs1800169 polymorphism in gene *CNTF* has been found to be associated with muscle strength in several studies. Walsh et al. reported a sex-specific effect in gene *CNTF* G/A polymorphism with only women of homozygous G alleles improving more in isometric elbow strength than A-allele carries after a 12-week upper arm training [41]. This is consistent with our results of the association between rs1800169 and dynamic knee contraction performance under a low load (∆PV_IT20_) with allele G exerting a favourable effect after the cessation of exercise. Yet, our findings contradict the cross-sectional study of De Mars et al. [42], who studied 493 adults (aged 38–80 years) and found that polymorphisms in gene *CNTFR* rather than *CNTF* were related to knee extension strength differences.

Although most of the data-driven SNPs identified in each muscular phenotype were different, we found eight SNPs that were related to multiple muscular phenotypes (Table S4). rs2251375 (from the *H19* gene) and rs1805087 (from the *MTR* gene) were associated with ∆PV_IT20_ and ∆PT_IK240_. rs3741211 (from the *IGF2* gene) and rs97713 (from the *MTRR* gene) were associated with ∆SMM and ∆PV_IT20_. rs327575 (from the *MTRR* gene) was associated with ∆PV_IM60_ and ∆PT_IK60_. rs4790881 (from the *SMG6* gene) was associated with ∆SMM and ∆PT_IK60_. rs2390760 (from the *METTL21C* gene) was associated with ∆SMM, ∆PT_IM60_ and ∆PT_IK60_. rs3762546 (from the *MSTN* gene) was associated with ∆SMM, ∆PT_IM60_ and ∆PT_IK240_. Therefore, these SNPs, which are broadly associated with muscular phenotypes, might be the focus of future gene studies in order to better understand muscle decline after the cessation of training.

### 4.3. Comparisons with Previous Longitudinal SNP Studies on Ageing Muscle

Generally, the one-year cessation of exercise in our study can be regarded as a one-year ageing process. Therefore, we compared our results with other genetic studies on long-term muscular changes related to ageing. A 5-year longitudinal study carried out by Delmonico et al. [43] among older adults aged 70–79 years found no significant association between *ACTN3* R577X (rs1815739) polymorphism and declined muscle strength in ageing. Another longitudinal study (mean follow-up period: 14.2 years) made by Schrager et al. [44] also showed that the *IGF2* ApaI (rs680) polymorphism was not related to losses of arm endurance capacity and grip strength. Similarly, these genes did not show significant linkage to muscular changes in our study. Yet, we did find seven common gene variants (Table S5), which were favourable for one-year exercise cessation-related muscular changes in the present study, that were previously reported in Charlier’s cross-sectional study on muscle mass and muscle strength among adults with an age range of 19–73 years [27] despite the fact that the favourable alleles (rs2854248 from the *ATP1A2* gene, rs3797297 from the *FST* gene and rs1801133 from the *MTHFR* gene) in three of these genes are not consistent with those in the present study. Such inconsistency might be related to the comparatively small sample size in both studies or the discrepancy in reference literature [27]. Therefore, further analyses are still needed to further confirm the predisposing alleles in genes related to muscle decline.

### 4.4. Limitations

The data-driven GPS is only one approach to investigate the association between one-year exercise cessation-related muscular decreases and gene variants. Many other processing methods such as total GPS, weighted GPS or elastic net GPS were also used in different studies with varied predictive powers. As found in the study of Charlier et al. [27], elastic net GPS had the best prediction on SMM while data-driven GPS and total GPS had the best prediction on strength-related phenotypes. Based on six genetic polymorphisms, Massidda et al. [25] found the weighted GPS explained more variance of explosive performances (18% squat jump and 24% counter-movement jump) than the total GPS. Noticeably, as presented in the first part of this discussion, data-driven GPS exerted similar predictive power regarding the muscular changes among the older participants after exercise training [23,24]. Therefore, for the consistency of approach in our previous study, we used a data-driven GPS approach in the present study.

Our conclusions are also limited considering the fact that the selection of data-driven SNPs and the predictive power of GPS were tested on the same sample. An application to an independent sample or cross-validation should better test our findings. Furthermore, GPS predictability depends on the selection and size of the initial SNP pool. Meanwhile, this is not a strict follow-up study on detraining since information of food consumption and exercise habit were not controlled. These factors might contribute to the non-significant changes in SMM and PT_IM60_ during the cessation period, which contradicts the findings of significant decreases in muscle strength and muscle among older participants 6 months after the cessation of a previous training study [45]. Notably, the estimation of SMM based on BIA (with a 9% standard error of estimate) might also contribute to the non-significant average change in SMM after the one-year of detraining. Therefore, one should be prudent when using the ∆SMM-driven SNPs identified in this study, and confirmations of those SNPs are needed through similar SNP analyses based on more accurate SMM measures (e.g., DEXA, MRI).

Since the genotyping in the current study was performed based on candidate-gene based muscle-related SNPs reported at an early time (up to August 2014), we failed to include gene variants that are identified by genome-wide association studies (GWAS) in recent years. For instance, a recent GWAS conducted by Hernandez Cordero et al. [26] identified 182 loci that were associated with appendicular lean mass, yet, those loci were also not included in our study. Zillikens et al. [46] also conducted a large-scale genome-wide association meta-analysis study on lean body mass and identified five loci (in or near the *HSD17B11*, *VCAN*, *ADAMTSL3*, *IRS1* and *FTO* genes) related to whole lean body mass. Although none of these loci were measured in our study, the metabolism-/proliferation-related genes (i.e., *IRS1* and *VCAN*) in Zillikens’ study suggested a possible functional connection with the metabolism-/growth-related genes (e.g., *ACVR1B*, *ATP1A1* and *IGF1*) identified in our study. Moreover, through the large-scale GWAS, Hernandez Cordero et al. [26] reported that SNP heritability explained 36% of the variance in lean mass while the identified 182 loci accounted for 24% of SNP heritability, indicating that the 182 loci could explain around 9% of the ALM variability. Zillikens el al. [46] also reported that all genotyped SNPs might explain 43.3%–44.2% of the lean body mass variance and each analysed replicated SNPs accounted for only 0.04% of the variance in whole lean body mass. While in our study, the data-driven GPS based on nine SNPs explained 27% of the variance in ∆SMM. The considerable proportion of ∆SMM variance explained by the small number of SNPs in our study might suggest that these identified SNPs are closely related to ∆SMM after the cessation of training. However, the small sample size in our study might also be very likely to inflate these explained variance proportions. Therefore, further studies on larger cohorts with more complete muscle-related loci will provide new insight into the genetic association with the loss of training benefit.

## 5. Conclusions

In this study, we applied stepwise regression and data-driven GPS methods from a 170-SNP set to explore the genetic effect on decreases in muscular phenotypes after one-year cessation of a structured training intervention. We found that GPSs accounted for 26% to 37% of the variances of corresponding muscular changes while participants with more favourable gene variants tended to have less declines in those changes. Moreover, 46 SNPs from 34 genes were identified to be significantly associated with these muscular alterations. These genes contribute to the domains of DNA methylation, metabolism, muscle growth, muscle structure and neural control. In addition, our results provide supportive explanations for the involvement of genetic variants in inter-individual variations of the loss of muscular benefits after the cessation of structured training among the older population.

## Figures and Tables

**Table 1:** Descriptive data and p values from ANOVA of between group comparisons at post-training and follow-up tests.

Parameters	Post-Training	Follow-Up	∆Follow-Post (%)	*p* Values from Repeated Measures ANOVA			
				Time	Time × Sex	Time × Group	Time × Sex × Group
AGE (year)							
FIT							
F	66.4 ± 3.8	-	-	-	-	-	-
M	67.5 ± 4.0	-	-				
WBV							
F	67.1 ± 5.2	-	-				
M	67.8 ± 4.5	-	-				
*p* value at Group level	0.55						
*p* value at Group × Sex level	0.84						
Height (m)							
FIT							
F	160.0 ± 7.9	-	-	-	-	-	-
M	174.3 ± 6.1	-	-				
WBV							
F	161.2 ± 5.7	-	-				
M	173.1 ± 6.5	-	-				
*p* value at Group level	0.99						
*p* value at Group × Sex level	0.28						
Body mass (kg)							
FIT							
F	66.6 ± 9.4	66.3 ± 8.9	−1.36 ± 2.80	<0.01 **	0.19	0.84	0.49
M	82.0 ± 9.6	83.2 ± 9.5	−0.09 ± 2.90				
WBV							
F	68.7 ± 9.3	68.6 ± 8.9	−0.17 ± 3.53				
M	79.0 ± 11.6	80.2 ± 12.8	−0.44 ± 3.21				
*p* value at Group level	0.77	0.85	0.97				
*p* value at Group × Sex level	0.15	0.16	0.61				
power at Group level	0.06	0.05	0.05				
power at Group × Sex level	0.30	0.29	0.08				
BMI (kg/m^2^)							
FIT							
F	26.1 ± 3.9	26.2 ± 3.8	−1.36 ± 2.80	<0.01 **	0.28	0.90	0.47
M	27.1 ± 3.3	27.4 ± 3.4	−0.09 ± 2.90				
WBV							
F	26.4 ± 3.5	26.5 ± 3.4	−0.17 ± 3.53				
M	26.4 ± 3.6	26.6 ± 3.6	−0.44 ± 3.21				
*p* value at Group level	0.79	0.68	0.97				
*p* value at Group × Sex level	0.41	0.39	0.61				
power at Group level	0.06	0.07	0.05				
power at Group × Sex level	0.13	0.14	0.08				
SMM (kg)							
FIT							
F	18.0 ± 2.0	17.7 ± 2.3	1.40 ± 8.29	0.45	0.96	0.55	0.83
M	30.2 ± 3.0	30.0 ± 3.1	4.21 ± 6.28				
WBV							
F	18.5 ± 2.2	18.5 ± 2.6	2.76 ± 9.52				
M	30.3 ± 3.2	30.8 ± 5.6	4.32 ± 17.25				
*p* value at Group level	0.58	0.29	0.53				
*p* value at Group × Sex level	0.76	0.97	0.95				
power at Group level	0.09	0.19	0.10				
power at Group × Sex level	0.06	0.05	0.05				
PT_IM60_ (Nm)							
FIT							
F	127.92 ± 18.18	127.79 ± 26.66	13.43 ± 17.70	0.43	0.93	0.64	0.64
M	186.32 ± 28.17	186.63 ± 32.58	16.50 ± 17.73				
WBV							
F	123.05 ± 27.56	125.63 ± 24.80	15.32 ± 18.18				
M	181.48 ± 36.61	174.20 ± 37.29	6.79 ± 22.37				
*p* value at Group level	0.41	0.31	0.76				
*p* value at Group × Sex level	1.00	0.48	0.64				
power at Group level	0.13	0.17	0.06				
power at Group × Sex level	0.05	0.11	0.08				
PV_IT20_ (°/s)							
FIT							
F	330.17 ± 37.73	307.58 ± 58.96	−1.63 ± 11.56	<0.01 **	0.39	0.68	0.67
M	377.62 ± 34.91	353.95 ± 35.29	−1.68 ± 9.37				
WBV							
F	328.08 ± 31.45	321.75 ± 33.10	0.87 ± 12.18				
M	364.79 ± 36.99	345.05 ± 40.26	−0.34 ± 15.02				
*p* value at Group level	0.29	0.78	0.65				
*p* value at Group × Sex level	0.45	0.22	0.85				
power at Group level	0.18	0.06	0.07				
power at Group × Sex level	0.12	0.24	0.05				
PT_IK60_ (Nm)							
FIT							
F	111.78 ± 17.98	102.65 ± 25.28	2.09 ± 6.79	0.02 *	0.25	0.27	0.56
M	168.54 ± 29.57	164.18 ± 30.00	5.70 ± 13.44				
WBV							
F	106.50 ± 18.50	107.98 ± 18.23	0.72 ± 8.9				
M	158.26 ± 28.67	156.29 ± 33.40	0.29 ± 17.16				
*p* value at Group level	0.12	0.84	0.14				
*p* value at Group × Sex level	0.61	0.30	0.71				
power at Group level	0.35	0.06	0.31				
power at Group × Sex level	0.08	0.18	0.07				
PT_IK240_ (Nm)							
FIT							
F	60.46 ± 10.26	53.11 ± 15.94	−0.83 ± 8.22	<0.01 **	0.97	0.50	0.85
M	93.58 ± 16.04	89.14 ± 14.63	3.76 ± 14.76				
WBV							
F	57.54 ± 10.45	57.24 ± 10.06	3.17 ± 10.19				
M	85.64 ± 14.62	82.28 ± 14.52	0.54 ± 16.63				
*p* value at Group level	0.04	0.66	0.32				
*p* value at Group × Sex level	0.34	0.08	0.51				
power at Group level	0.53	0.07	0.17				
power at Group × Sex level	0.16	0.41	0.10				

* *p* < 0.05, ** *p* < 0.01. Comparisons between the FIT and the WBV groups in muscular phenotypes were presented as *p* value and power at Group level, which showed that there was no significant difference (low *p* values and powers) between the two groups in most muscular phenotypes (except the PT_IK240_ at the post-training level). Moreover, no significant Group × Sex interaction was found in all muscular phenotypes, indicating that participants in both groups experienced similar changes in muscular phenotypes regardless of gender.

**Table 2:** Regressions of data-driven genetic predisposition scores (GPSs) and relative muscular changes after one-year cessation of structured training.

	GPS	SEX (M = 1, F = 0)	AGE	BMI	Corresponding Post-Training Value	Intercept	Adj. r^2^	NO. of SNPs
∆SMM (%)								
Estimate	2.09	−0.91	0.07	0.18	-	−29.36	0.27	9
β value	0.52	−0.07	0.05	0.09	-			
Partial r^2^	0.27	0.01	<0.01	0.01	-			
*p*	<0.01	0.39	0.58	0.27	-			
∆PT_IM60_ (%)								
Estimate	4.53	3.02	−0.02	0.50	−0.06	−38.69	0.32	7
β value	0.53	0.11	−0.01	0.12	−0.20			
Partial r^2^	0.27	0.01	<0.01	0.02	0.03			
*p*	<0.01	0.45	0.96	0.22	0.18			
∆PV_IT20_ (%)								
Estimate	2.24	1.90	−0.31	−0.09	−0.04	−3.40	0.40	13
β value	0.59	0.14	−0.18	−0.04	−0.22			
Partial r^2^	0.36	0.02	0.04	<0.01	0.06			
*p*	<0.01	0.22	0.08	0.66	0.05			
∆PT_IK60_ (%)								
Estimate	2.74	2.39	−0.16	0.23	−0.01	−19.44	0.37	9
β value	0.62	0.15	−0.08	0.09	−0.04			
Partial r^2^	0.37	0.01	0.01	0.01	<0.01			
*p*	<0.01	0.31	0.42	0.33	0.76			
∆PT_IK240_ (%)								
Estimate	2.56	0.84	−0.03	0.34	0.02	−68.75	0.27	18
β value	0.52	0.05	−0.01	0.12	0.04			
Partial r^2^	0.26	<0.01	<0.01	0.02	<0.01			
*p*	<0.01	0.78	0.90	0.23	0.78			

## Supplementary Materials

The following are available online at https://www.mdpi.com/2073-4425/11/9/968/s1, Table S1: The training programs, Table S2: 224 muscle-related SNPs, Table S3: Correlated SNPs and published references, Table S4: 170-SNP pool, Table S5: Common gene variants.
