# Supplementary material for: The Genetic Effect on Muscular Changes in an Older Population: A Follow-Up Study after One-Year Cessation of Structured Training
Source: Genes (Basel). 2020 Aug 21;11(9):968. doi: 10.3390/genes11090968 (PMC7564970; doi:10.3390/genes11090968)
Supplement: Supplementary file 1 [file genes-11-00968-s001.zip › LH_Table S5 Shared SNPs.pdf]

Table S5.1. Summary of common SNPs in the study of Charlier et al. (Charlier et al. 2017) and the present study

SNP	GENE	Muscular phenotype	Favourable allele	
			Charlier et al.	The present study
rs2854248	ATP1A2	Muscular strength, size, and trainability	A	T
rs3797297	FST	Muscle mass and strength	C	A
rs11549465	HIF1A	Muscular strength, size, and trainability	C	C
rs17727841	IGF1	Muscular strength, size, and trainability	C	C
rs689	INS	Muscular strength, size, and trainability	A	A
rs1801133	MTHFR	strength and training-related change	T	C
rs4253778	PPARa	Strength phenotypes	G	G

Charlier R, Caspers M, Knaeps S, et al (2017) Limited potential of genetic predisposition scores to predict muscle mass and strength performance in Flemish Caucasians between 19 and 73 years of age. Physiol Genomics 49:160–166 . doi: 10.1152/physiolgenomics.00085.2016
