# Supplementary material for: The Genetic Effect on Muscular Changes in an Older Population: A Follow-Up Study after One-Year Cessation of Structured Training
Source: Genes (Basel). 2020 Aug 21;11(9):968. doi: 10.3390/genes11090968 (PMC7564970; doi:10.3390/genes11090968)
Supplement: Supplementary file 1 [file genes-11-00968-s001.zip › LH_Table S1 Training Protocols.pdf]

Table S1.1. Detailed fitness training program

Period (week)	Strength Training Session: Sets (n)	Repetitions (n)	Intensity (% of 1 RM)	Goal[Δ]	Cardiovascular Training Session, % of heart rate reserve – duration (minutes): Monday	Wednesday	Friday
1→3	2	15	50	SE	70% - 2 × 10’	70% - 2 × 10’	70% - 2 × 10’
4→6	2	15	60	SE	70% - 20’ + 10’	80% - 20’	70% - 20’ + 10’
7→9	2	15	60-70	MS	70% - 2 × 15’	80% - 2 × 15’	70% - 2 × 15’
10→12	2	15	70	MS	80% - 30’	70% - 2 × 20’	80% - 30’
13→15	1	12	70-80	MS	70% - 35’	(70%-20’)+(80%-20’)	70% - 35’
16→18	2	12	70-80	MS	(80%-15’)+(70%-15’)+(80%-15’)	70% - 40’	(80%-15’)+(70%-15’)+(80%-15’)
19→21	1-2*	10	80	PO	70% - 2 × 20’	80% - 2 × 20’	70% - 2 × 20’
22→24	1	10	80	PO	80% - 30’ + 10’	(75%-20’)+(85%-20’)	80% - 30’ + 10’
27→29	1-2*	15	60-70	MS	70% - 2 × 20’	80% - 2 × 20’	70% - 2 × 20’
30→32	1	12	70-80	MS	80% - 30’ + 10’	(75%-20’)+(85%-20’)	80% - 30’ + 10’
33→35	2	12	70-80	MS	80% - 40’	(75%-20’)+(85%-20’)	80% - 40’
36→38	2	12	70-80	MS	(85%-20’)+(70%-15’)+(85%-15’)	75% - 40’	(85%-20’)+(70%-15’)+(85%-15’)
39→41	1-2*	8-10#	80	PO	(80%-20’)+(70%-10’)+(80%-15’)	80% - 40’	(80%-20’)+(70%-10’)+(80%-15’)
42→44	1-2*	8-10#	80	PO	80% - 2 × 20’	70% - 2 × 25’	80% - 2 × 20’
Post-tests (45→47)	1	8-10#	80	PO	(80%-20’)+(70%-10’)+(80%-15’)	80% - 40’	(80%-20’)+(70%-10’)+(80%-15’)

Participants were asked to train three times a week (Monday, Wednesday and Friday).
* Participants performed 2 sets on Monday and Friday. On Wednesday they performed 1 sets.
# Participants performed 10 repetitions on Monday and Friday. On Wednesday they performed 8 repetitions.
Δ SE: Strength Endurance; MS: Maximal Strength; PO: Power

Table S1.2. Detailed whole-body vibration training program

Period (week)	Volume: Duration of exercise (s)	Intensity: Frequency (Hz)	Intensity: Amplitude (High 5 mm/Low 2.5 mm)	Rest (s)	Modality	Number of series per exercise*: a	b	c	d	e	f	g	h
1→4	30	35	Low	60	static	3	1						
5→9	45	40	High	60	dynamic + static	3	1	1			1	1	
10→14	60	40	High	45	dynamic	3	3	3	1		1	1	
15→19	60	40	High	45	8-seconds principle#	3	3	3	1		1	1	
20→24	60	30	High	30	8-seconds principle#	3	3	3	1		1	1	
25→29	30-45	35	High	30	dynamic	3	3	3	1		1	1	
30→34	45-60	35	High	15	dynamic	3	3	3	2		1	1	1
35→39	60	35	High	15	8-seconds principle#	3	3	3	2	1	1	1	1
40→44	60	35	High	15	8-seconds principle# + dynamic	3	3	3	2	1	1	1	1
Post-tests (45→47)	60	35-40	High	15	8-seconds principle# + dynamic	2	2	2	1	1	1	1	1

* Lower Limb Exercise: a - squat (knee angle 90°), b - deep squat (knee angle 120 - 130°), c - wide stance squat, d - one legged squat, e - lunge, f - toes-stand, g - toes stand deep, h - moving heels.
# 8-seconds principle: 4 repetitions going slowly up (2 seconds) and down (2 seconds), 4 seconds static performance, 4 repetitions going up and down, static performance until the end of the exercise.
