# Supplementary material for: The Genetic Effect on Muscular Changes in an Older Population: A Follow-Up Study after One-Year Cessation of Structured Training
Source: Genes (Basel). 2020 Aug 21;11(9):968. doi: 10.3390/genes11090968 (PMC7564970; doi:10.3390/genes11090968)
Supplement: Supplementary file 1 [file genes-11-00968-s001.zip › LH_Table S2 Candidate genes and SNPs.pdf]

Table S2.1. Muscle-related candidate genes and SNPs

Gene abbreviation	Gene Name	Selected SNPs
**DNA methylation**		
MTHFR	methylene tetrahydrofolate reductase	rs1801131, rs1801133, rs4846048, rs1009592[+], rs1023252, rs11121828[+], rs12121543, rs12132479[+], rs12404124[+], rs13306553[+], rs13306556[+], rs1476413, rs1537516, rs17037396[+], rs17037425, rs17350396*, rs17421511[+], rs17421560[+], rs198375[+], rs198389[+], rs198391[+], rs198393[+], rs198405[+], rs198406[+], rs198411[+], rs198413*, rs198414[+], rs198415, rs1994798[+], rs2075538[+], rs2076001[+], rs2236797[+], rs4845882[+], rs4846052[+], rs4846054[+], rs535107[+], rs6540999[+], rs6541001[+], rs6541003[+], rs6668659[+], rs6676300, rs6697244[+], rs7538516[+], rs7553194[+]
MTR	5-methyltetrahydrofolate-homocysteine methyltransferase	rs1050993[+], rs10925235, rs1770449[+], rs1805087, rs4659725[+]
MTRR	5-methyltetrahydrofolate-homocysteine methyltransferase reductase	rs1801394, rs10475399, rs13166314[+], rs1532267, rs162031, rs162037*, rs162040, rs162048[+], rs2174147[+], rs2966952[+], rs2968311[+], rs326120[+], rs326123, rs327575, rs327588, rs329852, rs3733784[+], rs3776455, rs6866169[+], rs7703033, rs9313211, rs97713
**Growth/differentiation factor**		
ACVR1B	activin A receptor, type 1B	rs2854464, rs10783486, rs11612312, rs746434, rs10783485
ACVR2B	activin A receptor, type 2B	rs2268757
AKT1	v-akt murine thymoma viral oncogene homolog 1	rs1130214
CCL2	chemokine (c-c motif) ligand 2	rs1024610, rs1024611[+], rs13900[+], rs4586
CCR2	chemokine (c-c motif) receptor 2	rs1799865[+], rs3918358[+], rs768539
FST	follistatin	rs12152850, rs3797297

FoxO1	forkhead box O1	rs3751436[#]
H19	imprinted maternally expressed transcript (RNA-gene)	rs2251375, rs4929984
ID1	inhibitor of DNA binding 1	rs15817
ID3	inhibitor of DNA binding 3	rs11574
IGF1	insulin-like growth factor 1	rs17727841, rs35767
IGF2	insulin-like growth factor 2	rs3213221, rs3741211, rs3842759*
IGF2AS	insulin-like growth factor 2 antisense (RNA-gene)	rs7924316
IGFBP1	insulin-like growth factor binding protein 1	rs1065780
IGFBP3	insulin-like growth factor binding protein 3	rs2132570, rs2854744, rs3110697, rs6670
IL15	interleukin 15	rs1057972
IL15RA	interleukin 15 receptor, alpha subunit	rs2228059, rs2296135, rs3136618, rs7077401[+], rs8177586[+], rs8177600[+], rs8177613[+], rs8177636[+]
IL6	interleukin 6	rs1800795
INHBC	inhibin, beta C	rs2943693*, rs533975
MSTN	myostatin	rs1805086, rs3762546
MYOD1	myogenic differentiation 1	rs2526547, rs3911833*
MYOG	myogenin	rs4950877
PARD3	par-3 family cell polarity regulator	rs11009651
TGFB1	transforming growth factor, beta 1	rs1800470
TNF	tumor necrosis factor	rs1799964, rs1800630
**Hormone and receptor**		
ADRB2	β_2 adrenoreceptor	rs1042714[+], rs2053044[+], rs2400707
ADRB3	β_3 adrenoreceptor	rs4994
AR	androgen receptors	rs5919392, rs5965433

ESR1	estrogen receptor 1	rs1999805, rs4870044, rs1159327
INS	insulin	rs689
LEPR	leptin receptor	rs1137101, rs1045895, rs10493377, rs17412175, rs2025805, rs7602^{+}, rs9436302^{+}
RETN (RST)	resistin	rs1862513, rs3745367
TRHR	thyrotropin-releasing hormone receptor	rs16892496, rs7832552
VDR	vitamin D (1,25- dihydroxyvitamin D3) receptor	rs1544410, rs4516035, rs731236, rs7975232
**Metabolism**		
ACACB	acetyl-CoA carboxylase beta	rs2268388, rs2268405
ADAMTSL3	ADAMTS-like 3	rs899926
AMPD1	adenosine monophosphate deaminase 1	rs17602729*
APOA1	apolipoprotein A-I	rs12721026
ATP1A2	ATPase, Na^{+}/K^{+} transporting, alpha 2 polypeptide	rs1016732, rs2070704, rs2295623, rs2854248, rs3026468
BHMT	betaine-homocysteine s-methyltransferase	rs3733890
CACNA1S	calcium channel, voltage-dependent, L type, alpha 1S subunit	rs2296383, rs7415038
CKM	creatine kinase, muscle	rs8111989
CREBBP	CREB binding protein	rs129968
DNMT1	DNA (cytosine-5-)-methyltransferase 1	rs2241531
DNMT3A	DNA (cytosine-5-)-methyltransferase 3 alpha	rs2276598*
DNMT3B	DNA (cytosine-5-)-methyltransferase 3 beta	rs2424913
DNMT3L	DNA (cytosine-5-)-methyltransferase 3 like	rs7354779
HIF1A	hypoxia inducible factor 1, alpha subunit	rs11549465
KBTBD13	kelch repeat and BTB (POZ) domain containing 13	rs2919358

NOS3	nitric oxide synthase 3	rs1799983, rs2070744
NUAK1	NUAK family, SNF1-like kinase, 1	rs10861553*, rs3741886
PPARA	peroxisome proliferator-activated receptor alpha	rs4253778
PPP3R1	protein phosphatase 3, regulatory subunit b, alpha	rs3039851, rs687, rs6758449
PPP6R3	protein phosphatase 6, regulatory subunit 3	rs10896341
RYR1	ryanodine receptor 1 (skeletal)	rs2229139
**Muscle/bone structure**		
ACTN2	actinin, alpha 2	rs141416949[#], rs193922635[#]
ACTN3	actinin, alpha 3	rs1815739
BMP2	bone morphogenetic protein 2	rs15705, rs2145270, rs6107853
COL1A1	collagen, type I, alpha 1	rs1800012
FN1	fibronectin 1	rs10883631[+], rs7567647
GSC	goosecoid homeobox	rs4511463
METTL21C	methyltransferase like 21C	rs2390760
MYLIP	myosin regulatory light chain interacting protein	rs1011616
MYLK	myosin light chain kinase	rs2700352, rs28497577
OPN/SPP1	secreted phosphoprotein 1	rs28357094, rs10516796, rs11723364, rs13127257, rs1471403, rs1477602, rs1477603
SMG6	SMG6 nonsense mediated MRNA decay factor	rs4790881
TPM1	tropomyosin 1 (alpha)	rs707602
TTN	titin	rs10497520
**Neural Control**		
CNTF	ciliary neurotrophic factor	rs1800169
CNTFR	ciliary neurotrophic factor receptor	rs2070802, rs3808871
NTRK3	neurotrophic tyrosine kinase, receptor, type 3	rs2229910*

RIMS1	regulating synaptic membrane exocytosis 1	rs142196418
SORCS1	sortilin-related VPS10 domain containing receptor 1	rs4917481
NPY	neuropeptide Y	rs16139*
ZNF804A	zinc finger protein 804A	rs1483246
**Other**		
AGT	angiotensinogen	rs699
BTRC	beta-transducin repeat containing E3 ubiquitin protein ligase	rs10883642^{+}
CASP8	caspase 8, apoptosis-related cysteine peptidase	rs3769827
CCDC66	coiled-coil domain containing 66	rs6769055
GR/NR3C1	nuclear receptor subfamily 3, group C, member 1	rs6189*, rs6190
PPP1CC	protein phosphatase 1, catalytic subunit, gamma isozyme	rs1050587
UCP3	uncoupling protein 3	rs11235972, rs15763, rs1685354, rs1800849, rs647126

* Genotyping success rate <95%, $^{+}$ high linkage disequilibrium with other SNPs, $^{\#}$ SNPs with the same genotype in all participants
