# Supplementary material for: The Genetic Effect on Muscular Changes in an Older Population: A Follow-Up Study after One-Year Cessation of Structured Training
Source: Genes (Basel). 2020 Aug 21;11(9):968. doi: 10.3390/genes11090968 (PMC7564970; doi:10.3390/genes11090968)
Supplement: Supplementary file 1 [file genes-11-00968-s001.zip › LH_Table S3 Subgroups of high linkage disequilibrium.pdf]

Table S3.1 Subgroups of high linkage disequilibrium (LD)

Representative SNP	SNPs with high LD
rs1042714	rs1042714, rs2053044
rs10883631	rs10883631, rs10883642
rs17421511	rs12132479, rs17421511
rs13166314	rs13166314, rs3733784
rs1024611	rs13900, rs1024611
rs1799865	rs1799865, rs3918358
rs198389	rs198389, rs198375
rs1009592	rs6668659, rs1009592
rs7602	rs9436302, rs7602
rs198414	rs198411, rs198405, rs198414
rs1770449	rs1770449, rs4659725, rs1050993
rs2968311	rs2174147, rs2968311, rs6866169
rs326120	rs162048, rs326120, rs2966952
rs13306553	rs7553194, rs13306553, rs13306556, rs6541001, rs2075538
rs17421560	rs17421560, rs17037396, rs2236797, rs2076001
rs7077401	rs7077401, rs8177600, rs8177636, rs8177586, rs8177613
rs6697244	rs4845882, rs6697244, rs198393, rs535107
rs11121828	rs6540999, rs4846054, rs12404124, rs198391, rs11121828, rs6541003, rs198406, rs1994798
rs6541003	rs6541003, rs4846052, rs7538516
