# Supplementary material for: The Genetic Effect on Muscular Changes in an Older Population: A Follow-Up Study after One-Year Cessation of Structured Training
Source: Genes (Basel). 2020 Aug 21;11(9):968. doi: 10.3390/genes11090968 (PMC7564970; doi:10.3390/genes11090968)
Supplement: Supplementary file 1 [file genes-11-00968-s001.zip › LH_Table S4 Data-driven genes and SNPs.pdf]

Table S4.1. Summary of data-driven SNPs and genes

SNP	GENE	No. of SNPs in each gene	ΔSMM	ΔPT_{IM60}	ΔPV_{IT20}	ΔPT_{IK60}	ΔPT_{IK240}
rs10783485	ACVR1B	2					X
rs746434	ACVR1B						X
rs12721026	APOA1	1					X
rs1016732	ATP1A2	2					X
rs2854248	ATP1A2					X	
rs3733890	BHMT	1			X		
rs6107853	BMP2	1			X		
rs2296383	CACNA1S	1		X			
rs8111989	CKM	1		X			
rs1800169	CNTF	1			X		
rs4870044	ESR1	1	X				
rs10883631	FN1	1				X	
rs3797297	FST	1					X
rs4511463	GSC	1			X		
rs2251375	H19	1			X		X
rs11549465	HIF1A	1	X				
rs17727841	IGF1	1				X	
rs3741211	IGF2	1	X		X		
rs7924316	IGF2AS	1	X				
rs689	INS	1		X			
rs2919358	KBTBD13	1					X
rs1137101	LEPR	1					X
rs2390760	METTL21C	1	X	X		X	
rs3762546	MSTN	1	X	X			X
rs1009592	MTHFR	4					X
rs11121828	MTHFR				X		
rs1476413	MTHFR						X
rs1801133	MTHFR					X	
rs1805087	MTR	1			X		X
rs10475399	MTRR	8					X
rs162031	MTRR				X		
rs1801394	MTRR				X		
rs326123	MTRR						X
rs327575	MTRR			X		X	
rs7703033	MTRR					X	
rs9313211	MTRR						X
rs97713	MTRR		X		X		
rs4950877	MYOG	1					X
rs28357094	OPN/SPP1	1		X			
rs4253778	PPARa	1					X
rs142196418	RIMS1	1					X
rs2229139	RYR1	1	X				
rs4790881	SMG6	1	X			X	
rs1800470	TGFB1	1			X		

rs10497520	TTN	1				X	
rs1483246	ZNF804A	1			X		
SUM	34	46	9	7	13	9	18

Table S4.2. Summary of SNPs and genes associated with ΔSMM

SNP	GENE	No. of SNPs in each gene	GENOTYPE
rs4870044	ESR1	1	AA=2, AG=1, GG=0
rs11549465	HIF1A	1	CC=2, CT=1, TT=0
rs3741211	IGF2	1	CC=2, TC=1, TT=0
rs7924316	IGF2AS	1	GG=2, GT=1, TT=0
rs2390760	METTL21C	1	GG=2, CG=1, CC=0
rs3762546	MSTN	1	GG=2, CG=1, CC=0
rs97713	MTRR	1	CC=2, CT=1, TT=0
rs2229139	RYR1	1	GG=2, AG=1, AA=0
rs4790881	SMG6	1	AA=2, AC=1, CC=0
SUM	9	9	

Table S4.3. Summary of SNPs and genes associated with ΔPT_{IM60}

SNP	GENE	No. of SNPs in each gene	GENOTYPE
rs2296383	CACNA1S	1	TT=2, TC=1, CC=0
rs8111989	CKM	1	CC=2, TC=1, TT=0
rs689	INS	1	AA=2, AT=1, TT=0
rs2390760	METTL21C	1	GG=2, CG=1, CC=0
rs3762546	MSTN	1	GG=2, CG=1, CC=0
rs327575	MTRR	1	AA=2, AG=1, GG=0
rs28357094	OPN/SPP1	1	TT=2, GT=1, GG=0
SUM	7	7	

Table S4.4. Summary of SNPs and genes associated with ΔPV_{IT20}

SNP	GENE	No. of SNPs in each gene	GENOTYPE
rs3733890	BHMT	1	AA=2, AG=1, GG=0
rs6107853	BMP2	1	GG=2, AG=1, AA=0
rs1800169	CNTF	1	GG=2, AG=1, AA=0
rs4511463	GSC	1	GG=2, AG=1, AA=0
rs2251375	H19	1	AA=2, AC=1, CC=0
rs3741211	IGF2	1	TT=2, TC=1, CC=0
rs11121828	MTHFR	1	AA=2, AG=1, GG=0
rs1805087	MTR	1	AA=2, AG=1, GG=0
rs97713	MTRR	3	CC=2, TC=1, TT=0
rs1801394	MTRR		GG=2, AG=1, AA=0
rs162031	MTRR		CC=2, TC=1, TT=0
rs1800470	TGFB1	1	TT=2, TC=1, CC=0
rs1483246	ZNF804A	1	CC=2, TC=1, TT=0
SUM	11	13	

Table S4.5. Summary of SNPs and genes associated with ΔPT_{IK60}

SNP	GENE	No. of SNPs in each gene	GENOTYPE
rs2854248	ATP1A2	1	TT=2, AT=1, AA=0
rs10883631	FN1	1	AA=2, AG=1, GG=0
rs17727841	IGF1	1	CC=2, GC=1, GG=0
rs2390760	METTL21C	1	GG=2, CG=1, CC=0
rs1801133	MTHFR	1	CC=2, TC=1, TT=0
rs327575	MTRR	2	AA=2, AG=1, GG=0
rs7703033	MTRR		AA=2, AG=1, GG=0
rs4790881	SMG6	1	AA=2, AC=1, CC=0
rs10497520	TTN	1	TT=2, TC=1, CC=0
SUM	8	9	

Table S4.6. Summary of SNPs and genes associated with ΔPT_{IK240}

SNP	GENE	No. of SNPs in each gene	GENOTYPE
rs746434	ACVR1B	2	GG=2, AG=1, AA=0
rs10783485	ACVR1B		AA=2, AC=1, CC=0
rs12721026	APOA1	1	TT=2, TG=1, GG=0
rs1016732	ATP1A2	1	GG=2, AG=1, AA=0
rs3797297	FST	1	AA=2, AC=1, CC=0
rs2251375	H19	1	AA=2, AC=1, CC=0
rs2919358	KBTBD13	1	TT=2, TC=1, CC=0
rs1137101	LEPR	1	GG=2, AG=1, AA=0
rs3762546	MSTN	1	GG=2, GC=1, CC=0
rs1476413	MTHFR	2	GG=2, AG=1, AA=0
rs1009592	MTHFR		GG=2, GC=1, CC=0
rs1805087	MTR	1	AA=2, AG=1, GG=0
rs10475399	MTRR	3	AA=2, AG=1, GG=0
rs326123	MTRR		AA=2, AG=1, GG=0
rs9313211	MTRR		GG=2, AG=1, AA=0
rs4950877	MYOG	1	GG=2, AG=1, AA=0
rs4253778	PPARa	1	GG=2, GC=1, CC=0
rs142196418	RIMS1	1	CC=2, TC=1, TT=0
SUM	14	18	
